# Targeting Neurovascular Inflammation in Rosacea: A Systematic Review of Botulinum Toxin Therapy

**DOI:** 10.7759/cureus.111758

**Published:** 2026-06-29

**Authors:** Foteini Moniati, Chistos Costa, Constantina Chatzimatthaiou, Michaela Pishia, Maria Vassiliou, Marios Chatzimatthaiou

**Affiliations:** 1 Department of Inflammation and Aging, University of Birmingham, Birmingham, GBR; 2 Department of Medicine, Oxford University Hospitals NHS Trust, Oxford, GBR; 3 Department of Medicine, Medway NHS Foundation Trust, Gillingham, GBR; 4 Department of Medicine, Leeds Teaching Hospitals NHS Trust, Leeds, GBR; 5 Department of Medicine, Barts and the London School of Medicine and Dentistry, London, GBR

**Keywords:** botox, botulinum toxin, medical dermatology, rosacea, systematic review

## Abstract

Rosacea is a chronic inflammatory dermatosis characterized by persistent erythema and episodic flushing, often associated with suboptimal treatment outcomes and frequent recurrence. Botulinum toxin has recently been proposed as a novel therapeutic modality in its management. This systematic review evaluates the current evidence regarding the efficacy and safety of botulinum toxin in patients with rosacea. A comprehensive search of major electronic databases was conducted to identify relevant studies assessing its clinical application. Overall, the available literature suggests that botulinum toxin is associated with improvements in clinical signs and symptoms, particularly erythema, with effects sustained for a variable duration. Reported adverse events were predominantly mild and self-limiting. However, interpretation of these findings is limited by small sample sizes, heterogeneity in study design, and relatively short follow-up periods. Further high-quality, standardized studies are required to better define its role in routine clinical practice.

## Introduction and background

Rosacea is a chronic skin disorder that is characterized by a range of symptoms, including ocular manifestations, telangiectasia, papules, pustules, phymatous changes, and erythema [1]. Acknowledging the variety of clinical presentations, the Expert Committee of the National Rosacea Society developed a thorough categorization scheme in 2012 that distinguished four subtypes: erythematotelangiectatic, papulopustular, phymatous, and ocular rosacea [2]. Following this, a phenotype-based method emerged in 2017 to diagnose and classify rosacea, focusing on phymatous alterations and persistent centrofacial erythema as the main diagnostic criteria [3].

Treatment is primarily guided by the predominant clinical phenotype. For persistent erythema and telangiectasia, options include topical and oral agents, as well as laser and pulsed light therapies [4-5]. For papulopustular disease, topical and systemic anti-inflammatory and antimicrobial agents are favored, while phymatous changes are managed surgically or with ablative laser techniques [4-6]. Flushing remains the most difficult feature to treat, as most existing modalities target fixed erythema rather than the episodic vascular response [4]. More specifically, while topical treatments such as metronidazole, azelaic acid, sodium sulfacetamide, sulfur, and sodium sulfate offer localized action and reduce systemic exposure, they are limited by skin absorption challenges [5]. Concurrently, while doxycycline is the only Food and Drug Administration-approved oral therapeutic agent, other antibiotics, such as oral azithromycin, minocycline, and clarithromycin, are commonly used. Several drugs from other therapeutic classes, such as oral contraceptives, amitriptyline, clonidine, pimozide, aspirin, β-blockers, ondansetron, and COX-2 inhibitors, are also used to manage rosacea, each offering symptom relief through distinct mechanisms [4]. For severe or treatment-resistant rosacea, systemic agents such as oral isotretinoin, prednisolone, and ketoconazole have been used off-label, although evidence supporting their efficacy in this context remains limited [6]. Oral therapy, however, is associated with systemic side effects, while antibiotic administration raises significant concerns about bacterial resistance [5-6].

Furthermore, laser therapies, including vascular lasers and intense pulsed light, can be effective in reducing persistent erythema and visible telangiectasia. However, they are not effective in decreasing the frequency of flushing episodes. Lastly, for patients with phymatous rosacea, hypertrophic tissue can be sculpted and refined using ablative lasers, such as carbon dioxide lasers, or with electrosurgical tools [6].

Among the plethora of possible treatments, injectable neuromodulators such as botulinum toxin, type A, have shown promise in treating skin disorders and aesthetic flaws [7-9]. The usefulness of botulinum toxin in managing rosacea has been discussed in several reviews; nevertheless, the need for a thorough analysis has been highlighted by differences in study designs, formulations, dilutions, dosages, and outcome measures [10-11].

The rationale for botulinum toxin in rosacea is rooted in its proposed effects on neurovascular function. Rosacea is increasingly understood to involve dysregulation of cutaneous neurovascular signaling, whereby abnormal vasodilation, flushing, and persistent erythema are driven in part by the release of acetylcholine and vasoactive neuropeptides from cutaneous nerve fibers [3]. Botulinum toxin is thought to act by inhibiting this acetylcholine and neuropeptide release, thereby reducing vascular reactivity and inflammatory signaling implicated in flushing and erythema [3,8]. This proposed mechanism, distinct from its more established neuromuscular action, provides the biological basis for evaluating it as a treatment for the neurovascular features of rosacea and underpins the rationale for the present systematic review.

The objective of this article is to thoroughly examine all the published studies that look into the use of botulinum toxin for treating rosacea. The efficacy and safety profiles of this therapeutic approach will be thoroughly evaluated through a systematic review, providing insight into its potential as a viable treatment modality.

The preliminary findings of this study were reported as a conference abstract at EADV Symposium 2024 in St. Julian’s, Malta, in May 2024. This manuscript represents the complete systematic review, including comprehensive methodology, full results, and detailed discussion, and has not been published elsewhere.

## Review

Materials and methods

Protocol and Registration

No prospective protocol registration was undertaken for this systematic review.

Information Sources

A systematic review was carried out in accordance with the Preferred Reporting Items for Systematic Reviews and Meta-Analyses (PRISMA) guidelines, using both the PRISMA checklist and flow diagram [12]. The systematic search encompassed electronic databases, including PubMed, Embase, and the Cochrane Library, from inception to November 30, 2025, to identify studies on the use of botulinum in patients with rosacea.

Search Strategy

To ensure comprehensive identification of relevant studies, a comprehensive Medical Subject Headings (MeSH) search strategy was devised, incorporating the following universal search question: ("Botulinum" OR "Botox" OR "Botulinum Toxin") AND ("Rosacea"), along with its variations [13]. The full search strategy for each database, including Boolean operators, MeSH/Emtree terms, and applied filters, is provided in the Appendices section.

Eligibility Criteria

Eligibility was defined according to the PICOS framework. The population included patients diagnosed with rosacea of any subtype. Eligible interventions comprised the administration of botulinum toxin, irrespective of formulation, dose, dilution, or injection technique. No comparator was required, allowing the inclusion of single-arm prospective and retrospective studies, although studies employing control groups or split-face designs were also considered eligible. Outcomes of interest included any clinical, biophysical, patient-reported, or safety-related measures associated with rosacea, such as erythema, flushing, telangiectasia, quality of life, and adverse events. Eligible study designs included randomized controlled trials (RCTs), non-randomized prospective studies, retrospective studies, and case series. No minimum follow-up duration was specified owing to the limited and heterogeneous nature of the available evidence.

Only full-text articles published in English in peer-reviewed journals were included. Studies involving mixed populations were eligible provided that rosacea-specific outcomes were reported separately. Literature reviews, conference abstracts, animal studies, and registered trials without published results were excluded because they could not contribute extractable outcome data. Studies lacking full-text accessibility were also excluded to ensure accurate and consistent data extraction and risk-of-bias assessment across included studies.

Study Selection

Upon scrutinizing titles and abstracts, two evaluators (C.C. and M.C.) independently applied these criteria to select articles for further evaluation. Discrepancies between the reviewers were adjudicated by a third independent reviewer (F.M.). Subsequently, studies that met the eligibility criteria underwent a comprehensive full-text assessment.

Data Extraction

All retrieved articles underwent thorough assessment by two authors (C.C. and M.C.). Consensus on the inclusion or exclusion of studies was achieved unanimously. Instances of discordance prompted the involvement of a third reviewer (F.M.) to make the final decision. Following a predetermined protocol, we systematically collected data on primary authorship, study design details, sample sizes, demographic profiles of study participants, rosacea subtypes, commercial formulations, dosages, outcome measures, side effects, and follow-up details [13]. A data extraction template tailored specifically for this review was developed and underwent a pilot test, wherein it was applied to three randomly selected studies included in the review. Adjustments to the template were subsequently made based on insights garnered from this testing phase [13]. The responsibilities for data extraction and quality assessment were undertaken by two reviewers (C.C. and M.C.), with the accuracy of the compiled data validated by a third author (F.M.). Any potential discrepancies among the reviewing authors were resolved through a consensus-driven approach.

Risk-of-Bias Assessment

Methodological quality and risk of bias were assessed according to study design. The JBI Critical Appraisal Checklist was used for case series, as it is specifically designed to evaluate studies without comparator groups [14]. RCTs were assessed using the Cochrane Collaboration's tool, which examines domains including randomization, allocation concealment, blinding, incomplete outcome data, and selective reporting [15]. Non-randomized prospective studies were evaluated using the Methodological Index for Non-randomized Studies (MINORS), a validated instrument that assesses methodological quality in non-randomized designs, including prospective data collection, endpoint assessment, and the adequacy of control groups where applicable [16].

Each instrument was applied in accordance with its published guidance. For the JBI and MINORS tools, individual items were scored as 0 ("No"), 1 ("Unclear"), or 2 ("Yes"), and total scores were expressed as a percentage of the maximum achievable score. Studies were categorized as having a low, moderate, or high risk of bias if they achieved ≥80%, 60-79%, or <60% of the total score, respectively. Levels of evidence were additionally assigned according to the Oxford Centre for Evidence-Based Medicine 2011 Levels of Evidence [17]. To facilitate comparison across study designs, a numerical scoring approach was also applied to the Cochrane Collaboration's tool; however, as this instrument is primarily intended to provide domain-specific risk-of-bias assessments rather than an overall summary score, the resulting percentages should be interpreted as descriptive indicators of methodological quality rather than formal Cochrane risk-of-bias classifications.

Detailed tables can be accessed in the Appendices section. More specifically, it presents the Quality Risk of Bias Assessment, employing the MINORS, to evaluate five of the selected studies; the Quality Risk of Bias Assessment using the JBI Critical Appraisal Checklist, which was also applied to five of the selected studies; and the assessment of the remaining studies using the Cochrane Collaboration’s tool.

Synthesis Method

Given substantial heterogeneity in study design, botulinum toxin formulation, dosing, and outcome measures, a meta-analysis was not feasible. Instead, a narrative synthesis was conducted. Studies were grouped by outcome domain (erythema, flushing, quality of life, biophysical skin parameters, patient satisfaction, and adverse events). Within each domain, findings were synthesized by reporting the direction and, where available, the magnitude of change (percentage change from baseline) for each study. Where the original studies did not directly report percentage changes, these were calculated by the authors as [(post-treatment value − pre-treatment value) / pre-treatment value] × 100, using the means or values reported in each study; these calculated values are presented alongside extracted outcome measures and are treated as descriptive summaries of within-study change rather than pooled estimates. Safety outcomes were synthesized descriptively, categorizing adverse events as mild/self-limiting versus more notable events (e.g., transient facial motor dysfunction) and noting the frequency and resolution pattern of each.

Results

Study Characteristics

**Figure 1:**
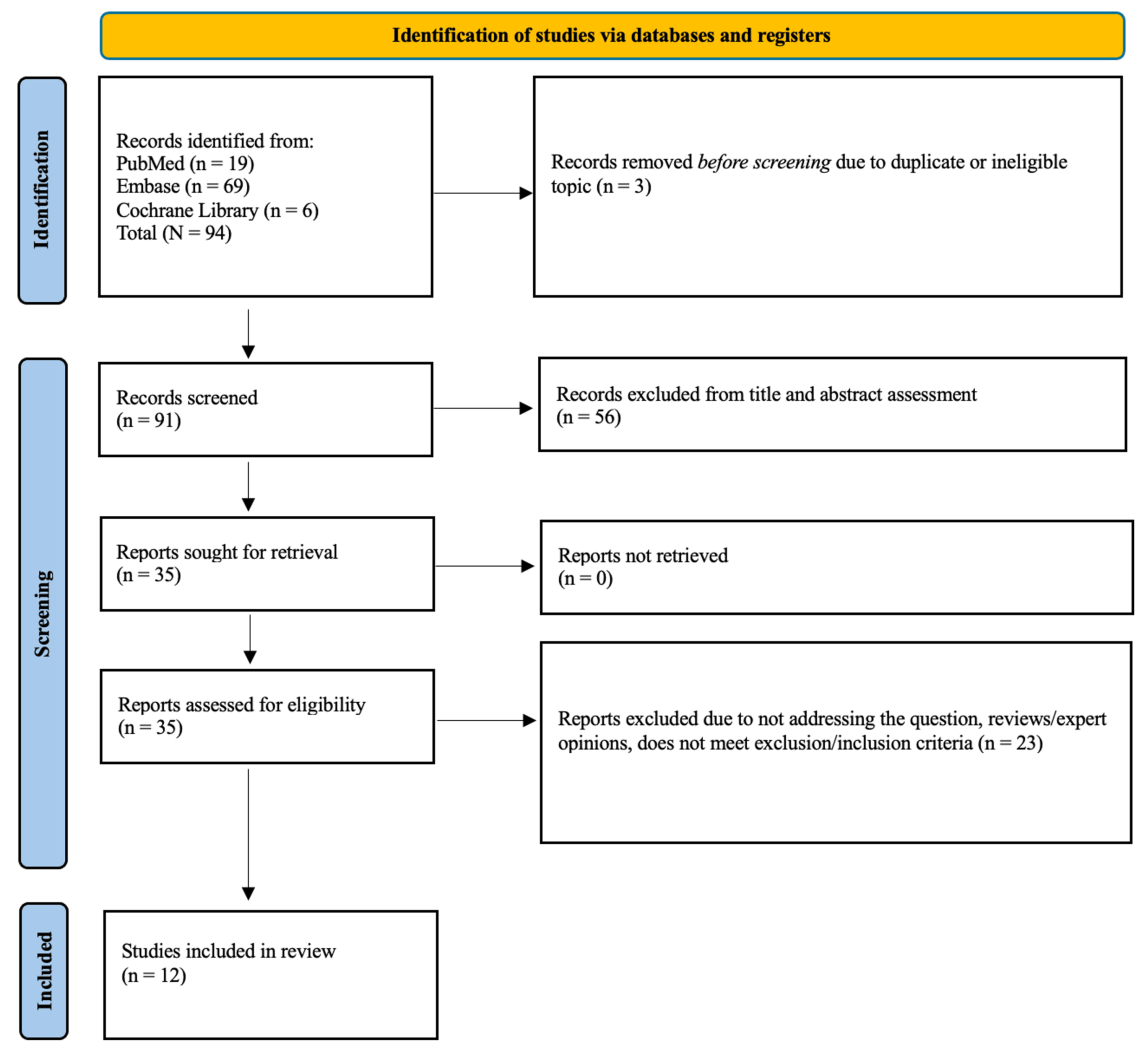
PRISMA flow diagram of the selected studies PRISMA: Preferred Reporting Items for Systematic Reviews and Meta-Analyses

Ninety-four studies were identified in the initial database search. After removing ineligible records by topic and those removed for other reasons, 91 records remained. Following abstract screening, 35 reports were assessed for eligibility, leaving 12 studies, published between 2012 and 2022, included in the final review. The search, conducted up to November 30, 2025, did not identify any additional eligible studies published after 2022 meeting our inclusion criteria. Among the examined studies, five were non-randomized prospective studies, five were prospective case series, and two were characterized as RCTs. The PRISMA flowchart, including reasons for exclusion, is illustrated in Figure 1.

The number of participants in each study ranged from 1 to 50, for a total of 212 participants across all studies included in this review. Out of the 12 studies, seven examined erythematotelangiectatic rosacea, two concentrated on erythematotelangiectatic and papulopustular rosacea together, and three did not determine the subtype of rosacea investigated. Botulinum toxin A was utilized in 12 studies, including six onabotulinumtoxinA, four abotulinumtoxinA, one incobotulinumtoxinA, and one prabotulinumtoxinA. Two studies did not include the formulation of botulinum toxin used. Dilution and dosing varied across studies.

All 12 studies yielded both qualitative and quantitative data from doctors and patients. The outcome of each study was assessed using various measures, including questionnaires, index values, and pre- and post-treatment photographs. For instance, Clinician Erythema Assessment (CEA), Rosacea Clinical Scorecard (RCS), Michaelsson Acne Score (MAS), and Global Flushing Severity Scale (GFSS) were all scale-based questionnaires used to assess the severity of rosacea-related symptoms, such as erythema and flushing. The degree of pain was specifically evaluated using the multifunctional test platform MPA10 (MultiProbe Adapter System; Courage + Khazaka electronic GmbH, Cologne, Germany) and the visual analog scale (VAS). Additionally, assessments such as the Patient Self-Assessment (PSA), State Self-Esteem Scale (SESS), Dermatology Life Quality Index (DLQI), and Global Aesthetic Improvement Scale (GAIS) assessed patients' feelings and satisfaction with the study treatment. Corneometer, mexameter, reviscometer, and sebumeter were used to provide biophysical measurements, and skin physiological indices were employed as quantitative measures to assess outcomes in a substantial portion of these studies. Such indices included Erythema Index (EI), transepidermal water loss (TEWL), sebum secretion, hydration, pH value, melanin content, and elasticity. Finally, Antera 3D cameras and VISIA scans were used to capture standardized images pre- and post-treatment, facilitating the measurement of erythema.

The duration of the post-treatment follow-up period, aimed at evaluating the aforementioned aspects, varied across studies, ranging from one week to nine months. The longest follow-up period, limited to nine months, may not provide sufficient time to comprehensively assess the treatment's long-term effects and potential adverse outcomes. Table 1 summarizes the baseline characteristics of the selected studies. Detailed dosing, dilution protocols, and outcome instruments for each study are provided in the Appendices.

**Table 1:** Summary of the baseline characteristics of selected studies CEA: Clinical Erythema Assessment, RCT: randomized controlled trial, N/A: not applicable

Author/year	Study design	No. of participants	Rosacea subtype	Follow-up
Yang et al., 2022 [18]	Pilot study, prospective	16	Erythematotelangiectatic	CEA at 1 month; flushing at 1, 3, 6 months; safety evaluation at 1, 3, 6 months
Calvisi et al., 2022 [19]	Clinical trial, prospective	50 (35 acne, 15 erythematotelangiectatic)	Erythematotelangiectatic	1, 2, 4 weeks and 4 months
Tong et al., 2022 [20]	Prospective, randomized, single blind, split-face controlled study (RCT)	22	Erythematotelangiectatic	Before treatment, 1, 2, 3, 6 months
Luque et al., 2021 [21]	Case series, retrospective	3	Erythematotelangiectatic and papulopustular	Not standardized - treatment efficacy mentioned in 3 weeks (case 3), 1 month (case 1, 2)
Al-Niaimi et al., 2020 [22]	Pilot study, prospective	20	Mostly Erythematotelangiectatic	3, 9 months
Friedman et al., 2019 [23]	Case series, retrospective	16	Erythematotelangiectatic and papulopustular	1, 3, 6 months
Kim et al., 2019 [24]	Randomized, double blind, split-face clinical study, prospective	24	Erythematotelangiectatic	2, 4, 8, 12 weeks
Bharti et al., 2018 [25]	Case, retrospective	1	N/A	4 months
Park et al., 2018 [26]	Pilot study, prospective	20	Erythematotelangiectatic	1, 2, 4, 8 weeks
Park et al., 2015 [27]	Case series, retrospective	2	N/A	3 months
Bloom et al., 2015 [28]	Noncontrolled single-center pilot study, prospective	25	Erythematotelangiectatic	3 months
Dayan et al., 2012 [29]	Case series, retrospective	13	N/A	3 months

Risk of Bias

The risk-of-bias assessment indicated a moderate-to-low overall risk of bias across the included studies. Among the five studies assessed using MINORS, two were rated low risk [18-19] and three moderate risk [22,27-28]. Of the five studies assessed using the JBI Critical Appraisal Checklist, three were rated low risk [23,26,29] and two high risk [21,25], the latter reflecting small sample sizes and limited reporting. Of the two RCTs assessed using the Cochrane Collaboration's tool, one was rated as moderate risk [20] and the other as high risk [24], primarily due to incomplete blinding and selective outcome reporting.

Efficacy Outcomes

Overall, the included studies demonstrated improvements in rosacea-related signs and symptoms following botulinum toxin treatment compared with baseline or pre-treatment assessments [18-29]. The most consistently reported improvements were reductions in erythema and flushing [18-23,27,29]. Improvement was also described in other rosacea-related features, including hydration, telangiectasia, pruritus, burning, edema, inflammation, and large pore size [20,22,25-26,29]. These changes were identified either through direct clinical observation or by quantitative outcome measures, including the CEA and EI [18,20,23-24,26].

Improvements in quality of life, assessed using the DLQI, were reported by Yang et al., Calvisi et al., and Friedman et al. [18-19,23]. Increased patient satisfaction or aesthetic improvement was also reported in several studies [19,22,24,26].

Safety Outcomes

As shown in Table 2, during the observational period, most adverse effects were mild and self-limiting [18-20,22-24,27-29]. The most frequently reported adverse effects were injection-related pain or discomfort [18-20,23,24,27,28], transient erythema [18,20,22,23], bruising [18-19,27], and edema [22-23]. These events were generally well tolerated and resolved spontaneously after treatment [18-20,22-23,27-28].

**Table 2:** Summary of the quantitative and qualitative findings of selected studies CEA: Clinician Erythema Assessment, DLQI: Dermatology Life Quality Index, sGAIS: Subject Global Aesthetic Improvement Scale, MAS: Michaelsson Acne Score, GFSS: Global Flushing Severity Score, EI: Erythema Index, TEWL: transepidermal water loss, PSA: Patient Self-Assessment, BTX: botulinum toxin

Author/year	Clinical outcomes	Adverse effects	Outcome measures	Key results
Yang et al., 2022 [18]	Improvement in flushing, CEA, and DLQI	Injection pain (all patients); transient erythema (n = 4, resolved within 20 minutes); bruising (n = 5, resolved within 1 week); facial tightness (n = 3, resolved within 1 month); transient asymmetry (n = 1, resolved within 1 month). No serious adverse events	CEA; flushing; DLQI	CEA: 2.88 ± 0.62 → 1.00 ± 0.37 (−65.28%). Flushing: 47.81 ± 8.68 → 26.50 ± 7.93 (−44.57%). DLQI: 22.25 ± 5.25 → 10.56 ± 3.53 (−52.54%)
Calvisi et al., 2022 [19]	Reduction in erythema and flushing; improved DLQI, skin quality, and sGAIS; acne subgroup improved	Mild pain and bruising, resolving within 1 week; no rosacea flares reported	DLQI; sGAIS; MAS	DLQI (rosacea): >54.66% improvement. sGAIS (rosacea): +3.46. MAS (acne): 55.48%
Tong et al., 2022 [20]	Improvement in erythema and hydration; reduction in GFSS, sebum secretion, VISIA red value, EI, and TEWL	Injection pain; transient erythema; mouth movement limitation (n = 1, resolved within 1 month)	EI; GFSS; TEWL; sebum; hydration; pH; VISIA red value	EI: 521.61 ± 33.97 → 428.55 ± 56.38 (−17.84%). GFSS: 7 (6.52-7.85) → 1 (0.89-1.93) (−85.71%). TEWL: 23.65 ± 4.56 → 10.48 ± 3.13 (−55.69%). Sebum: 46.87 ± 3.48 → 46.51 ± 2.67 (−0.77%). Hydration: 6.40 ± 6.25 → 47.09 ± 3.56. pH: 6.68 ± 0.08 → 6.69 ± 0.05 (0.15%). VISIA red value: 51.57 ± 6.18 → 41.06 ± 4.81 (−20.38%)
Luque et al., 2021 [21]	Reduction in erythema and flushing in all cases	Not reported	Clinical response	3/3 patients improved (100%); erythema/flushing reduction: 60-75%
Al-Niaimi et al., [22]	Improvement in erythema, flushing, telangiectasia, pruritus, and burning; high patient satisfaction	Reactive erythema, edema; mild purpura in one patient (resolved within 10 days)	Clinical response	20/20 patients improved (100%)
Friedman et al., 2019 [23]	Improvements in CEA, PSA, EI, and DLQI at 1, 3, and 6 months	Transient erythema, edema, discomfort, microcrusting; no motor deficits	CEA; DLQI; EI; PSA	CEA: 3.03 ± 1.10 → 1.81 ± 0.84 (−40.26%). DLQI: 18.6 ± 1.9 → 9.6 ± 2.8 (−48.39%). EI: 399.13 ± 119.23 → 299.62 ± 89.99 (−24.93%). PSA: 2.81 ± 0.93 → 1.87 ± 0.75 (−33.45%)
Kim et al., 2019 [24]	Reduction in CEA and EI; improvements in GAIS, hydration, and elasticity; no significant change in TEWL or sebum	Injection pain (tolerable)	CEA; EI; TEWL; sebum; hydration; melanin index; elasticity	CEA (BTX): 1.58 → 1.42 (−10.13%). CEA (control): 1.58 → 1.21 (−23.42%). EI (BTX): −4.87% ± 12.4. EI (control): −0.20% ± 12.9. TEWL: minimal change. Sebum: minimal change. Hydration: +7.26% ± 21.2. Melanin index: +6.02% ± 6.93. Elasticity: +9.21% ± 47.0.
Bharti et al., 2018 [25]	Reduction in erythema and edema lesions sustained over 3 months	Not reported	Clinical response	1/1 patient improved (100%)
Park et al., 2018 [26]	Reduction in erythema severity and EI; improved patient satisfaction	Injection pain; 3/20 patients developed unnatural facial expression (excluded)	EI; erythema severity; telangiectasia; satisfaction	EI (right cheek): 383.93 ± 63.82 → 304.71 ± 74.63 (−20.63%). EI (left cheek): 365.40 ± 60.42 → 308.82 ± 83.46 (−15.48%). Erythema severity: 1.90 ± 0.27 → 1.23 ± 0.48 (−35.26%). Telangiectasia: 1.65 ± 0.52 → 0.94 ± 0.44 (−43.03%). Satisfaction: 2.45 ± 0.54 → 2.94 ± 0.56 (+20%)
Park et al., 2015 [27]	Improvement in lesions and cosmetic outcomes	Mild transient injection discomfort and localized bruising	Clinical response	2/2 patients improved (100%)
Bloom et al., 2015 [28]	Improvement in erythema at 3 months	Minimal transient injection discomfort	Erythema grade	11/15 patients improved (73.33%). Erythema grade: 1.80 ± 0.56 → 1.00 ± 0.38 (−44.44%)
Dayan et al., 2012 [29]	Reduction in erythema, flushing, and inflammation within 1 week to 3 months	No adverse effects reported	Not reported	Not reported

More notable adverse effects included transient facial motor dysfunction in three studies [18,20,26]. Yang et al. reported a patient with an asymmetrical facial expression that resolved within one month [18]. Tong et al. described one patient with a temporary limitation of mouth movement that resolved within one month [20]. Park et al. reported three patients with unnatural facial expressions, which led to their exclusion from the study, with spontaneous resolution and no specific intervention required [26].

Discussion

Summary of Main Findings

Rosacea, a common, chronic inflammatory dermatosis, presents with a variety of clinical manifestations, including persistent erythema, paroxysmal flushing, pustules, papules, and telangiectasia, as well as ocular manifestations [30]. Botulinum toxin modulates neurovascular function by inhibiting acetylcholine and neuropeptide release, thereby ameliorating flushing, erythema, and telangiectasia in individuals with rosacea. Consequently, botulinum toxin is recommended for treating refractory paroxysmal flushing, moderate-to-severe persistent facial erythema, and rosacea unresponsive to standard therapeutic interventions [31].

In this comprehensive review of 12 studies, we found that botulinum toxin was effective in treating erythema and flushing in the erythematotelangiectatic and papulopustular subtypes of rosacea. Evidence regarding phymatous and ocular rosacea was limited. The studies demonstrated the efficacy of botulinum toxin in reducing the severity of erythema and flushing in patients with rosacea. The improvements were often quantifiable, as evidenced by reductions in clinical scores such as CEA, DLQI, and sGAIS. An observable improvement was evident within one to two weeks post-injection, and the positive effects lasted for three to six months. After this duration, certain patients experienced a return of rosacea symptoms, albeit with reduced intensity compared to baseline, necessitating additional injection sessions. Unfortunately, an assessment of the recurrence rate following the initial injection could not be conducted due to a lack of precise numbers regarding patients with recurring symptoms in the available studies.

Clinical Interpretation

According to Alexis et al., the worldwide prevalence of rosacea in people with skin of color varies, with rates up to 10% and estimates of 40 million cases [32]. However, rosacea might be underdiagnosed and underreported in populations with darker skin color due to the challenge of discerning telangiectasia and erythema [32]. Furthermore, Alexis et al. note that patients with skin of color who have rosacea experience delayed diagnosis, inadequate treatment, uncontrolled disease progression, and greater rates of morbidity [32].

Apart from skin color, rosacea manifestations vary with Fitzpatrick skin type [33-35]. While persistent erythema is the commonest symptom in skin types I-IV, early identification of erythema in patients with types V or VI is challenging, as the initial noticeable symptoms are papules and pustules. The literature selected for our systematic review spans multiple countries, including the United States, China, and India. Although some studies lack explicit descriptions of Fitzpatrick skin types, individuals with pale white skin are typically classified as type I or II. The spectrum of skin types among Asians encompasses Chinese types III and IV to Indian types IV and V [36]. Consequently, this review incorporates a broader range of skin types, serving as a valuable resource for clinicians working with patients of diverse racial backgrounds and skin tones.

Mechanistic Rationale

Notably, a combination of genetic and environmental factors is implicated in the incidence and pathogenesis of rosacea, including, but not limited to, alcohol consumption, smoking, ultraviolet radiation, and age [30]. Concurrently, dysregulated immune system activity, along with dysfunction in the immune (e.g., mast cells), nervous, and cardiovascular systems, significantly contributes to the pathophysiology of this condition [37]. The disease process involves the intricate interplay of numerous mediators and pathways, with previous studies substantiating the roles of vascular abnormalities and inflammation.

According to Woo et al., increased expression of toll-like receptor 2 (TLR2) contributes to the pathogenesis of rosacea [38]. TLR2 activation stimulates keratinocytes to release kallikrein 5, a key protease involved in disease development [39]. Kim et al. further demonstrated elevated levels of antimicrobial peptides in affected individuals [40]. The active peptide LL-37 binds to TLR2, triggering inflammatory signaling pathways and upregulating pro-inflammatory biomarkers, while also activating the NLRP3 inflammasome, a major contributor to cutaneous inflammation [41-42]. Mast cells also play an important role by releasing mediators that promote vasodilation, angiogenesis, and tissue fibrosis [43].

Lee et al. highlighted the association between angiogenesis and chronic inflammatory skin diseases, demonstrating that LL-37 promotes pathological angiogenesis in rosacea through microbial- and mast cell-mediated vascular endothelial growth factor signaling [44]. Animal studies have shown that botulinum toxin can inhibit TLR2-mediated inflammation, reduce LL-37-induced erythema, and suppress mast cell degranulation, thereby attenuating skin inflammation [45-46]. Collectively, these findings suggest that botulinum toxin may exert therapeutic effects in rosacea by inhibiting acetylcholine and neuropeptide release and modulating TLR2, LL-37, and mast cell activity, ultimately reducing inflammation and pathological angiogenesis.

Limitations

Our systematic review also has limitations. Foremost among these limitations is the small sample size across the reviewed studies, with six of 12 having fewer than 20 patients. This diminishes the likelihood of obtaining statistically significant results and conducting power calculations effectively. Simultaneously, due to the limited availability of high-quality RCTs assessing the effectiveness of botulinum toxin in rosacea management, we included case series, reports, and non-randomized controlled studies, thereby increasing the risk of bias. Additionally, there was inconsistency in follow-up duration across the included studies, with some investigations spanning only a month. Given the recurrent nature of rosacea, a relatively brief follow-up period may distort an accurate assessment of the efficacy of botulinum toxin treatment. This limitation arises from the challenge of sustaining treatment efficacy over time and the risk of excessively bolstering clinicians' confidence. As the majority of included studies did not report P-values or 95% confidence intervals for within- or between-group comparisons, the percentage changes summarized in this review are descriptive. They should not be interpreted as statistically validated effects.

Furthermore, although the studies included in our analysis provided a more extensive representation of skin color types, none directly compared treatment efficacy across patients with diverse skin color types. This raises uncertainty regarding potential variations in the effective treatment dose and the maintenance of efficacy across different skin color types. Lastly, the varied reporting of measured outcomes across the studies rendered a meta-analysis of efficacy impractical.

Future Research Direction

The use of botulinum toxin for rosacea remains an off-label intervention, with no established recommendations regarding optimal dilution protocols, injection techniques, dosing regimens, or treatment intervals. The heterogeneity in botulinum toxin formulations and administration methods reported across the included studies presents an additional challenge for clinical implementation. Furthermore, variability in treatment durability and the lack of guidance on maintenance therapy highlight the need for standardized treatment protocols and clearer reporting of dilution ratios and procedural details. Although the pathogenesis of rosacea involves complex interactions between TLR2, LL-37, kallikrein 5, mast cells, lymphocytes, and vasoactive neuropeptides, the precise mechanisms through which botulinum toxin exerts its therapeutic effects remain incompletely understood. Further mechanistic studies and high-quality clinical trials are therefore needed to clarify its role and inform evidence-based recommendations for the use of botulinum toxin in rosacea.

## Conclusions

This systematic review provides a comprehensive and up-to-date overview of the use of botulinum toxin in the treatment of rosacea. Current evidence suggests that botulinum toxin has acceptable efficacy and safety in treating rosacea. However, the findings are constrained by factors such as small sample sizes, imperfect study designs, and short follow-up periods. While serving as a foundational systematic review, this study may lay the groundwork for future research and prove valuable in decision-making regarding the use of botulinum toxin for rosacea treatment.

It is therefore imperative to conduct larger, randomized, placebo-controlled studies with extended follow-up periods to address these limitations. Determining the optimal dilution and dosing is crucial, contingent upon the severity of symptoms. Further research should explore therapeutic effects across different formulations and rosacea subtypes, while incorporating appropriate outcome measurements. Additionally, there is a need for in-depth investigations into the molecular mechanisms of botulinum toxin in the treatment of rosacea while assessing the efficacy of combination therapies involving botulinum toxin.

## Appendices

Table 3 shows the full search strategy for each database, including Boolean operators, Medical Subject Headings (MeSH)/Emtree terms, and applied filters. Table 4 presents the Quality Risk of Bias Assessment, employing the Methodological Index for Non-randomized Studies (MINORS), to evaluate five of the selected studies. Table 5 presents the Quality Risk of Bias Assessment using the JBI Critical Appraisal Checklist, which was also applied to five of the selected studies. Table 6 presents the assessment of the remaining studies using the Cochrane Collaboration’s tool. Finally, Table 7 summarises the commercial botulinum toxin formulations, dosing regimens, dilution protocols, and outcome measures reported across the included studies.

**Table 3:** Full electronic search strategy and results for the systematic review

Database	Search string	Filters/limits	Number of results
PubMed	("Botulinum Toxins"[MeSH Terms] OR "botulinum"[Title/Abstract] OR "botox"[Title/Abstract] OR "botulinum toxin"[Title/Abstract] OR "botulinum toxin type A"[Title/Abstract] OR "onabotulinumtoxinA"[Title/Abstract] OR "abobotulinumtoxinA"[Title/Abstract] OR "incobotulinumtoxinA"[Title/Abstract] OR "prabotulinumtoxinA"[Title/Abstract]) AND ("Rosacea"[MeSH Terms] OR "rosacea"[Title/Abstract] OR "erythematotelangiectatic rosacea"[Title/Abstract] OR "papulopustular rosacea"[Title/Abstract])	English language; Humans; Full text available; database inception to 30 November 2025	19
Embase	('botulinum toxin'/exp OR 'botulinum toxin a'/exp OR botulinum:ti,ab OR botox:ti,ab OR 'botulinum toxin':ti,ab OR onabotulinumtoxina:ti,ab OR abobotulinumtoxina:ti,ab OR incobotulinumtoxina:ti,ab OR prabotulinumtoxina:ti,ab) AND ('rosacea'/exp OR rosacea:ti,ab OR 'erythematotelangiectatic rosacea':ti,ab OR 'papulopustular rosacea':ti,ab)	English language; Humans; database inception to 30 November 2025	69
Cochrane Library (CENTRAL)	#1 MeSH descriptor: [Botulinum Toxins] explode all trees; #2 (botulinum OR botox OR "botulinum toxin"):ti,ab,kw; #3 MeSH descriptor: [Rosacea] explode all trees; #4 (rosacea):ti,ab,kw; #5 (#1 OR #2) AND (#3 OR #4)	Database inception to 30 November 2025	6

**Table 4:** MINORS N/A: not applicable, MINORS: Methodological Index for Non-randomized Studies

Author/year	Clearly stated aim	Inclusion of consecutive patients	Prospective collection of data	Endpoint appropriate to aim of study	Unbiased assessment of the study	Follow-up period appropriate to aim	Loss to follow-up less than 5%	Prospective calculation of the study size	Adequate Control group	Contemporary groups	Baseline equivalence of groups	Adequate statistical analysis	Total	Percentage conversion	Risk of Bias Status
Yang et al., 2022 [18]	2	2	2	2	2	2	2	2	N/A	N/A	N/A	N/A	16 out of 16	100%	Low
Calvisi et al., 2022 [19]	2	2	2	2	2	2	2	0	N/A	N/A	N/A	N/A	14 out of 16	87.50%	Low
Al-Niaimi et al., 2020 [22]	1	1	1	2	1	2	2	0	N/A	N/A	N/A	N/A	10 out of 16	62.50%	Moderate
Park et al., 2015 [27]	2	1	2	2	0	2	0	2	N/A	N/A	N/A	N/A	11 out of 16	68.75%	Moderate
Bloom et al., 2015 [28]	2	1	2	2	0	2	0	2	N/A	N/A	N/A	N/A	11 out of 16	68.75%	Moderate

**Table 5:** JBI Critical Appraisal Checklist N/A: not applicable

Author/year	Was a consecutive or random sample of patients enrolled?	Was a case-control design avoided?	Did the study avoid inappropriate exclusions?	Were the index test results interpreted without knowledge of the results of the reference standard?	If a threshold was used, was it pre-specified?	Is the reference standard likely to correctly classify the target condition?	Were the reference standard results interpreted without knowledge of the results of the index test?	Was there an appropriate interval between the index test and the reference standard?	Did all patients receive the same reference standard?	Were all patients included in the analysis?	Total	Percentage Conversion	Risk of bias status
Luque et al., 2021 [21]	0	0	2	1	N/A	2	1	0	2	2	10 out of 18	55.56%	High
Friedman et al., 2019 [23]	2	0	2	2	2	2	2	2	2	2	18 out of 20	90%	Low
Bharti et al., 2018 [25]	0	0	1	1	N/A	2	1	2	N/A	N/A	7 out of 14	50%	High
Park et al., 2018 [26]	1	0	2	2	N/A	2	2	2	2	2	15 out of 18	83.33%	Low
Dayan et al., 2012 [29]	1	0	1	2	2	2	2	2	2	2	16 out of 20	80%	Low

**Table 6:** Cochrane Collaboration’s tool

Author	Random sequence generation	Allocation concealment	Blinding of participants and personnel	Blinding of outcome assessment	Incomplete outcome data	Selective reporting	Other bias	Total out of 14	Percentage conversion	Risk of bias status
Tong et al., 2022 [20]	1	1	2	2	2	2	0	10 out of 14	71.43%	Moderate
Kim et al., 2019 [24]	1	1	2	2	1	1	0	8 out of 14	57.14%	High

**Table 7:** Detailed characteristics of botulinum toxin interventions and outcome measures in reported in studies BTX: botulinum toxin, BTX-A: botulinum toxin type A, CEA: Clinician Erythema Assessment, DLQI: Dermatology Life Quality Index, sGAIS: Subject Global Aesthetic Improvement Scale, GAIS: Global Aesthetic Improvement Scale, MAS: Michaelsson Acne Score, GFSS: Global Flushing Severity Score, EI: Erythema Index, TEWL: transepidermal water loss, VAS: Visual Analog Scale, PSA: Patient Self-Assessment, VISIA: Canfield facial imaging system, MPA10: MultiProbe Adapter system

Author/year	Commercial botox name	Dosing and dilution	Measured outcomes
Yang et al., 2022 [18]	N/A	100U BTX-A added to 6.25 mL sterile 0.9% NaCl to achieve a concentration of 16 U/mL BTX-A solution. 0.05 mL of (0.8U) BTX-A solution was injected at each point in the erythema area; a total of 40-60 U of BTX-A was used in each patient	CEA score (used facial photographs using VISIA Canfield imaging system, modified questionnaire of flushing symptoms, DLQI, safety evaluation
Calvisi et al., 2022 [19]	OnabotulinumtoxinA - microbotox	Botulinum toxin solution was reconstituted from a 50 Botox Units (UB) vial of onabotulinumtoxinA using 0.75 mL saline plus 0.5 mL lidocaine (0.5%) without adrenaline, for a total volume of 1.25 mL. 0.5 mL (20 UB) was drawn from this solution, and 0.5 mL of saline or lidocaine (0.5%) without adrenaline was added to bring the total to 1 cc for injection into the face	Erythema and flushing, DLQI, sGAIS, MAS
Tong et al., 2022 [20]	OnabotulinumtoxinA	Each bottle of 100 U BTX is dissolved in 2.5 mL normal saline and diluted to 1 U/0.1 mL. 0.05-0.1 mL was injected at each point; the injection depth was about 0.5-1 mm. Depending on the severity and area, the injection volume for the unilateral cheek ranges from 10 to 15 U	GFSS, VISIA red value, skin physiological indexes (EI, TEWL, sebum secretion, hydration, and pH value) using the multi-functional test platform MPA10 (MultiProbe Adapter System), VAS for pain degree evaluation
Luque et al., 2021 [21]	AbotulinumtoxinA and incobotulinum	3.75U per 0.1 mL (abobotulinum) and 1.25U per 0.1 mL (incobotulinum) were used for each patient (dosing was variable in patients)	Vascular signs using polarized photography and symptoms of rosacea
Al-Niaimi et al., 2020 [22]	AbotulinumtoxinA and onabotulinumtoxinA	500 units in a volume of 5 mL, 20 to 50 units used per cheek (abobotulinumtoxinA), 2.5 mL dilution in 100 units, 10 to 20 units per cheek (onabotulinumtoxinA)	Standardized photographs were taken prior and post treatment using Antera 3D camera for erythema quantification, CEA
Friedman et al., 2019 [23]	AbotulinumtoxinA	100 U in 3 mL of bacteriostatic saline	CEA, DLQI, Mexameter, PSA score
Kim et al., 2019 [24]	PrabotulinumtoxinA	Diluted with injectable normal saline, 1 U/ 0.1 mL, 15 U of BTX administered in one cheek	CEA score, GAIS score, TEWL, skin hydration, melanin content, erythema index, elasticity, and sebum secretions
Bharti et al., 2018 [25]	N/A	BTX diluted to 10 U/mL, 0.05 mL microdroplet injections	Vascular symptoms using dermoscopy
Park et al., 2018 [26]	OnabotulinumtoxinA	50 U of BTX-A with human serum albumin and NaCl reconstituted with 2.5 mL saline. Final concentration of 2 U/0.1 mL achieved, 20 U for each patient	Severity of erythema and telangiectasia by a non-treating investigator on a 4-point scale, EI by mexameter (MX18), patient satisfaction using 5-point scale
Park et al., 2015 [27]	OnabotulinumtoxinA	50 U of BTX type A with human serum albumin and NaCl reconstituted with 2.5 mL saline. Final concentration of 2 U/0.1 mL achieved, 50 U administered via two treatments	Cosmetic/aesthetic results
Bloom et al., 2015 [28]	AbotulinumtoxinA	Every 300 units were reconstituted with 3 mL bacteriostatic NaCl 0.9%. Intradermal injections of 15-45 units in affected areas	Facial erythema assessment by a nontreating investigator using a standardized grading system (standardized digital photographs)
Dayan et al., 2012 [29]	OnabotulinumtoxinA	Intralesional injections in a dilution of 7cc saline solution/100 U, 8-12 units per affected area	Rosacea symptoms (erythema and flushing)
